# Template switching and duplications in SARS-CoV-2 genomes give rise to insertion variants that merit monitoring

**DOI:** 10.1038/s42003-021-02858-9

**Published:** 2021-11-30

**Authors:** Sofya K. Garushyants, Igor B. Rogozin, Eugene V. Koonin

**Affiliations:** National Center for Biotechnology Information, National Library of Medicine, National Institutes of Health, Bethesda, MD USA

**Keywords:** Genome informatics, SARS-CoV-2

## Abstract

The appearance of multiple new SARS-CoV-2 variants during the COVID-19 pandemic is a matter of grave concern. Some of these variants, such as B.1.617.2, B.1.1.7, and B.1.351, manifest higher infectivity and virulence than the earlier SARS-CoV-2 variants, with potential dramatic effects on the course of the pandemic. So far, analysis of new SARS-CoV-2 variants focused primarily on nucleotide substitutions and short deletions that are readily identifiable by comparison to consensus genome sequences. In contrast, insertions have largely escaped the attention of researchers although the furin site insert in the Spike (S) protein is thought to be a determinant of SARS-CoV-2 virulence. Here, we identify 346 unique inserts of different lengths in SARS-CoV-2 genomes and present evidence that these inserts reflect actual virus variance rather than sequencing artifacts. Two principal mechanisms appear to account for the inserts in the SARS-CoV-2 genomes, polymerase slippage and template switch that might be associated with the synthesis of subgenomic RNAs. At least three inserts in the N-terminal domain of the S protein are predicted to lead to escape from neutralizing antibodies, whereas other inserts might result in escape from T-cell immunity. Thus, inserts in the S protein can affect its antigenic properties and merit monitoring.

## Abstract (web summary)

Sofya Garushyants et al. survey publicly available alignments of SARS-CoV-2 genomes to identify and characterize potential insertion variants throughout the COVID-19 pandemic. They altogether identify 347 inserts, some of which might affect SARS-CoV-2 pathogenicity.

## Introduction

The first SARS-CoV-2 genome was sequenced in January 2020. Since then, more than a million virus genomes have been collected and sequenced. Comparative analysis of SARS-CoV-2 variants has provided for the identification of the routes of virus transmission^1–4^, the selective pressure on different genes^5^, and the discovery of new variants associated with higher infectivity^6–8^. In many cases, genome analysis only included search for point mutations, but some deletions also have been identified, such as del69-70, one of the characteristic mutations of B.1.1.7 and Cluster 5^2,3^ or del157-158 in B.1.617.2 (delta)^9^. Moreover, recently, recurrent deletions have been shown to drive antibody escape^10^. However, insertions are mostly ignored, both during variant calling step and in the downstream analysis.

Although insufficiently studied, insertions appear to be crucial for beta-coronavirus evolution. Three insertions in the spike (S) glycoprotein and in the nucleoprotein (N), that occurred early in sarbecovirus evolution, have been shown to differentiate highly pathogenic beta-coronaviruses (SARS-CoV-1, SARS-CoV-2 and MERS) from mildly pathogenic and non-pathogenic strains, and suggested to be key determinants of human coronaviruses pathogenicity^11^. The best characterized insert in SARS-CoV-2 is the PRRA tetrapeptide that so far is unique to SARS-CoV-2 and introduces a polybasic furin cleavage site into the S protein, enhancing its binding to the receptor^12–14^.

Inserts in the SARS-CoV-2 genome are categorized in the CoV-GLUE database^15^, and the preliminary results on systematic characterization of the structural variation and inserts in particular have been reported^16^. Forty structural variants including three inserts, three nucleotides long each, were discovered and shown to occur in specific regions of the SARS-CoV-2 genome. These variants have been further demonstrated to be enriched near the 5’ and 3’ breakpoints of the non-canonical (nc) subgenomic (sg) RNAs of coronaviruses. In addition, indels have been shown to occur in arms of the folded SARS-CoV-2 genomic RNA^16^. However, longer inserts that might have been introduced into the virus genome during SARS-CoV-2 evolution, to our knowledge, have not been systematically analyzed.

The mechanisms of sequence insertion in the genomes of RNA viruses, and coronaviruses in particular, are poorly understood. One potential route is recombination. Homologous recombination is common among coronaviruses, and in particular, in the sarbecovirus lineage, and is likely to be a major evolutionary route producing coronavirus strains with changed properties^17,18^. Specifically, the entire receptor-binding motif (RBM) domain of the S protein can be replaced by homologous recombination as it probably happened in RaTG13 and some other sarbecoviruses^17,19,20^. In contrast, non-homologous recombination in RNA viruses appears to be rare, and its molecular mechanisms remains poorly understood^21^.

In infected cells, beta-coronaviruses produce 5–8 major sgRNAs^22,23^. Eight canonical sgRNAs are required for the expression of all encoded proteins of SARS-CoV-2. These sgRNAs are produced by joining the transcript of the 5′ end of the genome (TRS site) with the beginning of the transcripts of the respective open reading frames (ORFs)^24^

In addition, SARS-CoV-2 has been reported to produce multiple nc sgRNAs, some of which include the TRS at 5′ end, whereas others are TRS-independent^25,26^; apparently, the ncRNAs are spurious products of errors of transcription initiation.

Here we report the comprehensive census of the inserts that were incorporated into virus genomes during the evolution of SARS-CoV-2 over the course of the pandemic and show that they occurred during virus evolution rather than resulting from experimental errors. These inserts are non-randomly distributed along the genome, most being located in the 3′terminal half of the genome and co-localizing with 3′ breakpoints of nc-sgRNAs. We show that at least some long insertions occur either as a result of the formation of nc-sgRNAs or by duplication of adjacent sequences. We analyze in detail the inserts in the S glycoprotein and show that at least three of these are located in a close proximity to the antibody-binding site in the N-terminal domain (NTD), whereas others are also located in NTD loops and might lead to antibody escape, and/or T cell evasion.

## Results

### Identification of inserts in SARS-CoV-2 genomes

**Fig. 1:**
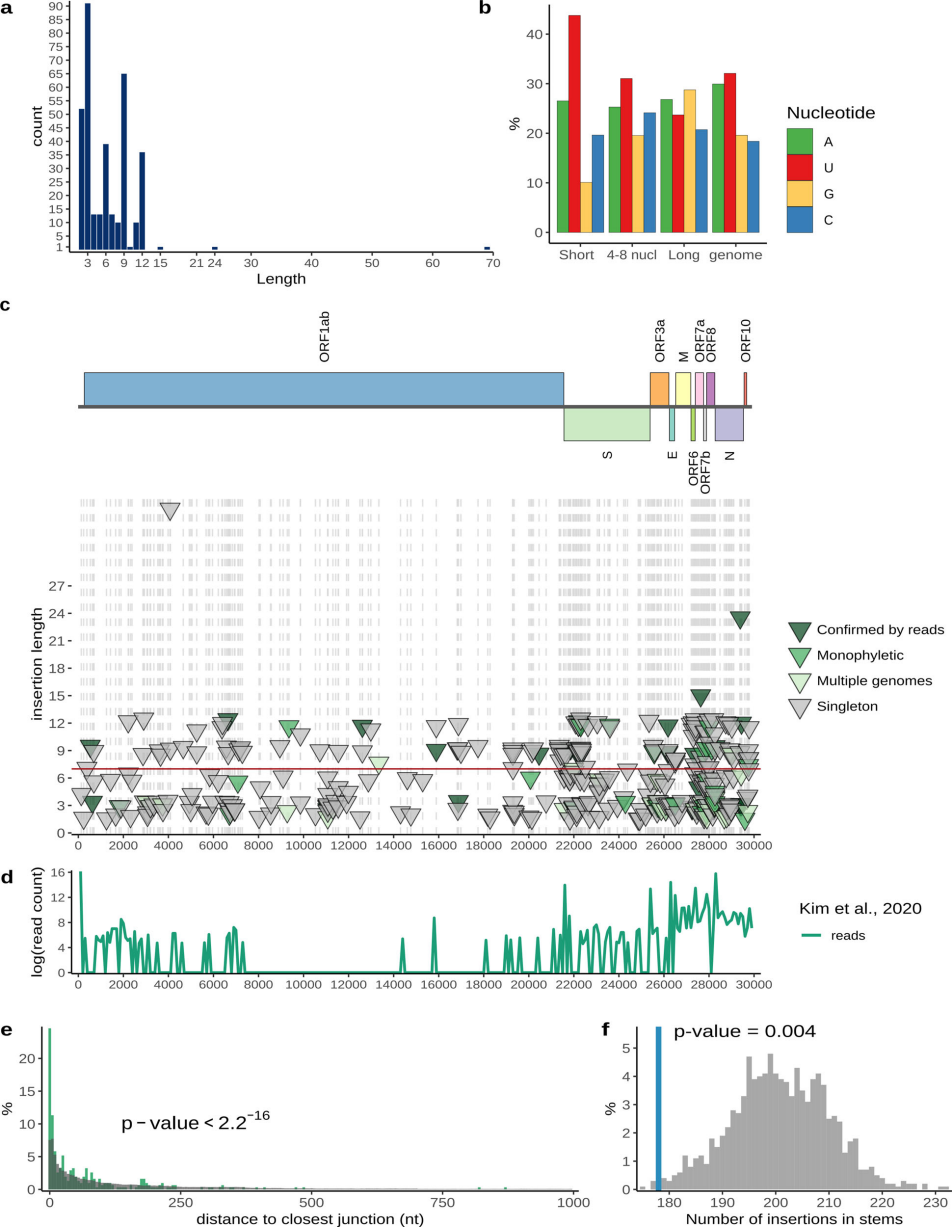
Insertions in SARS-CoV-2 genomes. **a** Distribution of insert lengths. **b** Nucleotide composition of inserts of different lengths and full SARS-CoV-2 genome. **c** Distribution of inserts along the genome. Each triangle represents one insertion event. The level of confidence in each variant is represented by color: dark green, confirmed by sequencing read analysis; green, monophyletic in the tree, no read data available; light green, observed multiple times, but not monophyletic; gray, singletons (Supplementary Data 2). The positions of inserts are marked with gray dashed lines. **d** Experimental data on SARS-CoV-2 transcriptome^26^ showing template switch hotspots during the formation of sgRNAs, showing the distribution of junction reads connecting recombination hotspots along the genome. **e** Distance from inserts to closest template switching hotspot site (green) compared with random expectation (gray). Wilcoxon rank sum test *p* value is provided. **f** The number of inserts that occur in structured regions of SARS-CoV-2 genomic RNA (blue) compared with random expectation (gray). Permutation test *p* value is provided. The data on SARS-CoV-2 structure was obtained from^28^. The code to reproduce the figure is provided in repository (see Code availability).

To compile a reliable catalogue of inserts in SARS-CoV-2 genome, we analyzed all the 1,785,103 sequences present in the GISAID multiple genome alignment (compiled on June 17, 2021). From this alignment, we extracted all sequences that contained insertions in comparison with the reference genome (1354 unique events in 2159 unique genomes). After the initial filtering (Methods), insertions were identified in 752 unique genomes, with 544 unique events detected in total. We evaluated all regions around insertions in alignments and removed all inserts that appeared due to misalignment. To the remaining inserts, we added four long inserts obtained from the GISAID metadata description (see Methods), resulting in a set of 354 unique inserts ranging in length from 2 to 69 nucleotides in 746 genomes (including identical sequences) (Fig. 1a; Supplementary Data 1; Supplementary Data 2).

To further minimize the number of inserts that appeared due to sequencing errors, we screened the Sequence Read Archive (SRA) database for the corresponding raw read data. We were able to obtain raw reads for 43 inserts, of which 40 were multiples of three by length, and one more was four nucleotides long but occurred in an intergenic region. We validated 35 insertions with raw read data; only one of these was not a multiple of three in length and occurred within a gene (Supplementary Data 1; Supplementary Data 2). Among the inserts that were not validated by raw reads 6 were singletons, whereas two others were short duplets and occurred in a polyU tract. We removed these unconfirmed events from our dataset, resulting in 346 unique inserts. Assuming that the fraction of true positives is the same among all inserts as it is among those with available reads, 282 of these inserts are expected to reflect actual evolutionary events. Among the inserts in our dataset, 234 (67%) were multiples of three, and of the remaining ones, 16 (5% of the total) were located in intergenic regions, 39 (11%) in orf1ab, and 57 (17%) in other genes. It appears likely that most if not all frameshifting inserts are sequencing artifacts, but some of such inserts in other genes could be real events reflecting the dispensability of these genes for virus reproduction. For example, we identified four frameshifting inserts in ORF6, for which deletion variants have been described earlier^27^

The short inserts (<9 nt) had a distinct nucleotide composition with a substantial excess of uracil, at about 45%, whereas the composition of the long inserts (9 nt or longer) was similar to that of the SARS-CoV-2 genome average, with about 25% U (Fig. 1b). This trend was even more pronounced for inserts verified by raw reads (Supplementary Fig. 1). Thus, we split the collection of inserts into two categories, the short and long inserts, which we analyzed separately.

We then checked whether inserts that were present in multiple genome sequences were located close to each other in the global phylogenetic tree (see Methods). We did not require strict monophyly because inserts are not always included in the genome sequences (see Supplementary Note 1; Supplementary Fig. 2, Supplementary Fig. 3). Of the 52 short inserts identified in multiple genomes, 18 met this relaxed monophyly criterion (Supplementary Data 2). In five cases, identical insertions were observed in genomes submitted from the same laboratory, and mostly, on the same date, which implies that the genomes were sequenced and analyzed together, making it difficult to rule out a sequencing error. Interestingly, seven of the eight cases that were validated by raw reads were not monophyletic. By contrast, among the 15 long inserts that were found in multiple genomes, 14 met the monophyly criteria including all 5 validated by raw reads (Supplementary Data 2).

In summary, the inserts detected in SARS-CoV-2 genomes fell into the following categories: 230 (66.5%) short inserts, among which 15 (4.3% of the total) were validated by raw reads; and 116 long (at least, 9 nt) (33.5%) inserts. We additionally classified the long inserts into four groups, in the order of increasing confidence: 87 (25%) singletons, 1 non-monophyletic insert observed in two genomes, 9 (2.6%) monophyletic inserts observed in multiple genomes, all with no available raw reads and 20 (5.8%) inserts (15 singletons and 5 monophyletic ones), for which the insertions were validated by the raw reads. The 15 (4.3%) short inserts confirmed by read data and 29 (8.4%) long inserts that were detected in multiple genomes (monophyletic and not) and/or confirmed by raw reads comprised the set of the most reliable insertion events that were observable across the evolution of SARS-CoV-2 during the pandemic (Supplementary Data 3). Among these highly reliable inserts, there was only one frameshifting insert within orf6 gene.

### Insertions are non-uniformly distributed along the SARS-CoV-2 genome

We found that the insertions were not randomly distributed along the genome, with most occurring in the 3′-terminal third of the genome (Fig. 1c). Two, not necessarily mutually exclusive main hypotheses have been proposed on the origin of the short inserts (structural variants) in coronavirus, namely, that they are associated with loops in the virus RNA structure or occur near the hotspots of template switch, at the breakpoints of TRS-independent transcripts^16^. To differentiate between these two mechanisms, we compared the distribution of 347 inserts along the SARS-CoV-2 genome with the distributions of structured regions^28^ and of template switch hotspots^26^. We detected a strong association of the insertions with the template switch hotspots (Pearson correlation *r* = 0.42, *p* value = 1.8 × 10^−14^) (Fig. 1d). Almost 25% of the inserts occurred within 5 nt of a template switch hotspot, compared to <10% expected by chance, and the distribution of lengths observed in the real data is significantly different from random expectation (Wilcoxon test *p* value < 2.2 × 10^−16^; Fig. 1e). Also, we observed that inserts were significantly less frequent in predicted RNA stems than expected by chance (permutation test *p* value = 0.004; Fig. 1f). Both these observations held, with statistical significance, when we analyzed only the 45 highly confident insert (Supplementary Fig. 4). Thus, inserts in SARS-CoV-2 genomes are associated with template switch hotspots, and also tend to occur in RNA loops.

### Short insertions in SARS-CoV-2

The notable difference in nucleotide composition and different phyletic patterns of short and long inserts imply that the two types of insertions occur via different molecular mechanisms. As pointed out above, the short insertions are rarely monophyletic, indicating that short U-rich sequences were inserted in the same position in the SARS-CoV-2 genome on multiple, independent occasions during virus evolution in the course of the pandemic. Also, 43 of the 230 short inserts occurred in runs of U or A, whereas 63 more probably result from local duplications (Supplementary Data 2). These observations suggest that short insertions occur via template sliding (polymerase stuttering) on runs of As or Us in the template (negative strand or positive strand, respectively) RNA^29–31^ (Supplementary Fig. 5a; Supplementary Data 2). This could be either a biological phenomenon occurring during SARS-CoV-2 evolution, in case the errors are produced by stuttering of the coronavirus RdRP, or an artifact if the errors come from the reverse transcriptase or DNA polymerase that is used for RNA sequencing, or are a mix of biological and experimental polymerase errors. However, for all 17 short inserts that were confirmed by raw read analysis, we also detected the U enrichment (Supplementary Fig. 1). Those inserts were observed at high allele frequencies in the data (Supplementary Data 1), and thus, are unlikely to result from experimental errors. In addition, short inserts appear to be represented with the same frequency in SARS-CoV-2 genomes sequenced with different technologies, including Illumina MiSeq, NovoSeq and NextSeq, and even Oxford Nanopore or IonTorrent (Supplementary Data 1). Furthermore, elevated rate of thymine insertion has not been reported as a common error of either Illumina or Oxford Nanopore technology^32–35^. In contrast, production of longer transcripts and slow processing on polyU tracts has been demonstrated for nsp12 (RdRP) of SARS-CoV-1^36^. In addition, the RdRp complex of SARS-CoV lacking the proof-reading domain has been shown to misincorporate more nucleotides compared with other viral polymerases^37^. Thus, it appears likely that most of the short inserts in SARS-CoV-2 genomes are generated by stuttering of the virus RdRP.

### Molecular mechanisms of long insertions

**Fig. 2:**
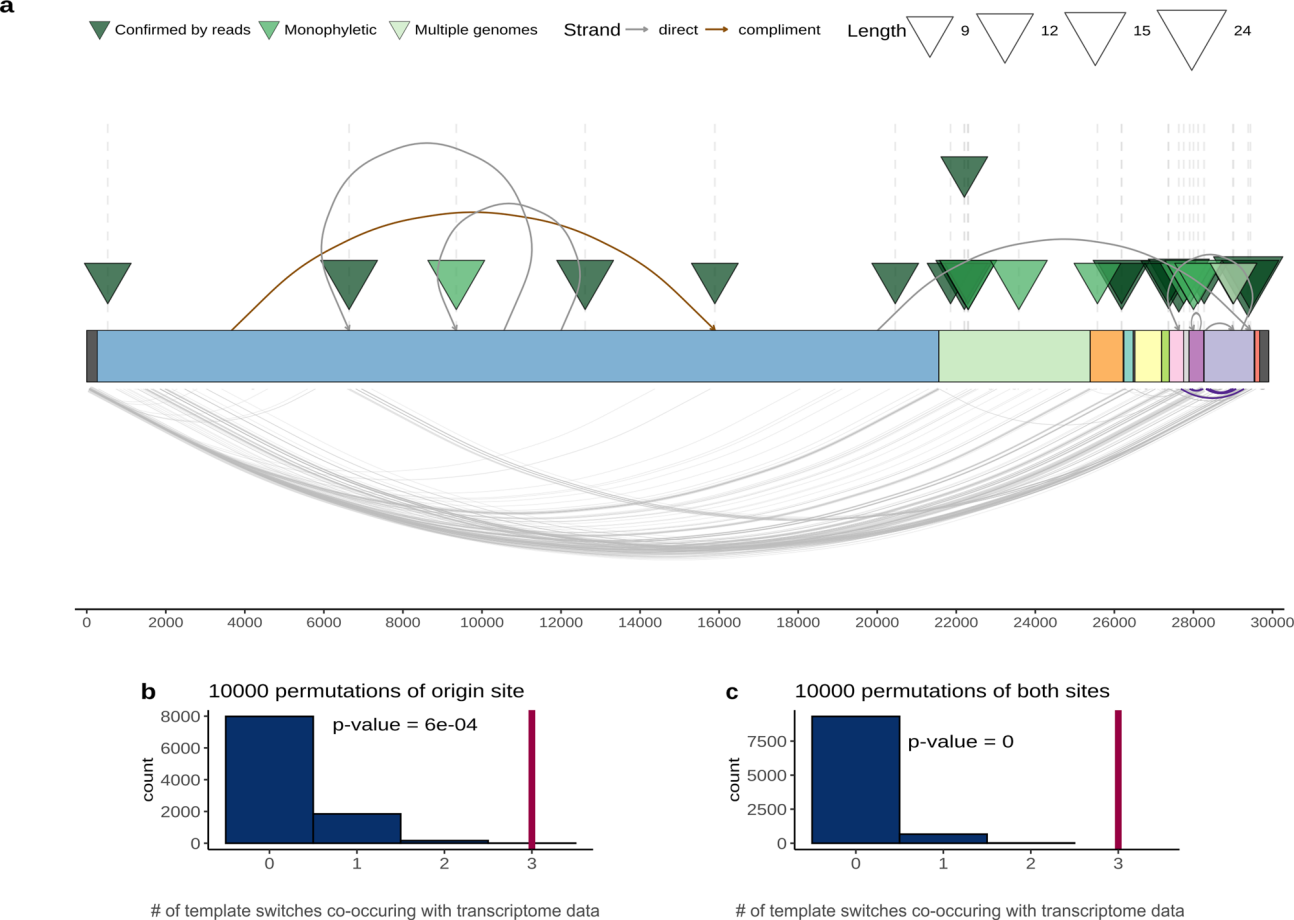
Long insertions possibly occur through template switching and formation of nc sgRNAs. **a** Each triangle shows an independent insertion event, colored as in Fig. 1. Curves on the upper side of the plot connect the insertion origin site and insertion position, brown color indicates that the origin sequence is on the same strand, and gray color shows that the origin sequence is on complementary strand, curves at the bottom of the plot represent the experimental data on sgRNAs from Kim et al.^26^. Curves highlighted in violet correspond to the three cases when insert and corresponding origin site co-occur with sgRNA junctions. The SARS-CoV-2 genes are colored as in Fig. 1. Permutation tests show the number of template switches co-occurring with RdRp jumps (*x*-axis) expected at random (blue), (**b**) when only the positions of the origins were randomly sampled 10,000 times from the genome; **c** when both ends were randomly sampled. Red vertical line represents the number observed in data. The code to reproduce the permutation tests is available in repository (see Code availability).

For in-depth analysis of the long inserts, we selected only the 29 high-confidence ones (see above), which were found in 74 genomes and ranged in size from 9 to 24 nucleotides (Fig. 2, Supplementary Data 4).

Those insertions originated from different laboratories that employed different protocols. Furthermore, these events started to accumulate in late April 2020, and the median collection date of the genomes containing the long inserts is February, 8 2021. Eight of the 29 reliable long insertions are in the S gene, which is significantly more than expected by chance (Binomial test *p* value = 0.027). The excess of inserts in the S gene suggests that their spread in the virus population could be caused by positive selection, perhaps, driven by enhancement of the interactions of SARS-CoV-2 with the host cells conferred by the inserts. This possibility is in agreement with the recent detection of an excess of amino acid replacements apparently due to positive selection in the S gene ^38,39^, although a contribution of relaxed purifying selection cannot be ruled out either.

The lengths of these high-confidence inserts allowed us to search for matching sequences both in SARS-CoV-2 genomes and in other viruses. For nine cases, we were able to identify the probable origin of the insertion. All detected matches were within the SARS-CoV-2 genome and contained no substitutions. For two inserts, we detected a local duplication that most likely gave rise to the insertion (Supplementary Fig. 5b). In both cases, these inserts were found in multiple genomes and are probably monophyletic, and one of these inserts was validated with raw read data. In seven more cases, we detected significant matches in the SARS-CoV-2 genome, six in the coding strand and one in the complementary strand (Fig. 2a; Supplementary Data 4). Among these seven insertions, two were monophyletic, and four more were singletons supported by raw data. The apparent origin of inserts from distant parts of the SARS-CoV-2 genomes implies template switch (Supplementary Fig. 5c). In three more cases, we were able to propose possible sources of inserts in SARS-CoV-2 genome, but the matches did not reach statistical significance (Supplementary Data 4). Although we could not find a probable source for many inserts, which is a limitation of our analysis, the principal reason for this limitation is that most inserts are only 9 nt long, so that there is not enough statistical power for detecting likely origin sequences if they contain any substitutions.

We hypothesized that the long inserts, at least those, for which potential origin sequences were detected, originate from template switching that occurs during the synthesis of the nc sgRNAs, when the RdRP jumps from one genome location to another. To assess the possibility that RdRP jumping contributes to insertions, we compared the insert locations and the sites of the likely origin of the inserts with the experimental data on the SARS-CoV-2 transcriptome^26^. The jumps produce junction reads that connect two distant locations in the genome. Regions that are connected by multiple reads are hotspots of template switching (Fig. 2). If the insertion sequence and the origin sequence are located close to these junction sites, it is likely that they were introduced by the RdRP during sgRNA synthesis. As pointed out above, inserts show a non-random proximity to template switch hotspots, so for the inserts with a traceable origin, we additionally checked whether their sites of origin occurred close to the sites of RdRp jumping. Although the information on the SARS-CoV-2 transcriptome is limited, among the seven inserts with predicted origins, we found that three insert sites were located within one end of the junction, whereas their corresponding sites of origin were within 100 nucleotides of the other side of the same junction (Fig. 2a). To assess the significance of this finding, we performed two permutation tests (see Methods), in one of which the real insertion positions were matched with start sites chosen randomly, whereas in the second one, both types of sites were selected at random. Both tests showed significant co-localization of the inserts with template switch junctions (Fig. 2b, c).

Thus, high-confidence long inserts in the SARS-CoV-2 genome probably originated either by local duplication or by template switching which, at least in some cases, seemed to be associated with nc sgRNA synthesis. Notably, the PRRA insert, the furin cleavage site that is one of characteristic features of SARS-CoV-2, resembles the long inserts analyzed here. Although this insert has a high GC-content compared to the genomic average of SARS-CoV-2, it falls within the GC-content range of the long inserts (Supplementary Fig. 1b). Furthermore, this insert is located within 20 nucleotides of a template switch hotspot at position 22,582^26^.

### Insertions in the S protein yield putative antibody escape variants

As indicated above, insertions are non-uniformly distributed along the SARS-CoV-2 genome (Fig. 1с). In particular, among the 29 long inserts identified with high confidence, 8 were located in the S protein, suggesting that these inserts could persist due to their adaptive value to the virus. Four of the 8 inserts in S were observed in multiple genomes that formed compact clades in the phylogenetic tree (Supplementary Note 1), and two (ins214AAG and ins214TDR) were strongly supported by raw reads. Four more inserts (ins98KAE, ins214KLGP, ins245IME, and ins246DSWG) were found in single genomes, but again, were strongly supported by raw reads, where they reached allele frequency close to unity, so these are highly unlikely to be artifacts (Supplementary Data 1). Inserts in the S protein were observed in various PANGO lineages, and furthermore, ins246DSWG occurred in B.1.1.7 (Alpha) lineage (Supplementary Data 4; Supplementary Note 2).

**Fig. 3:**
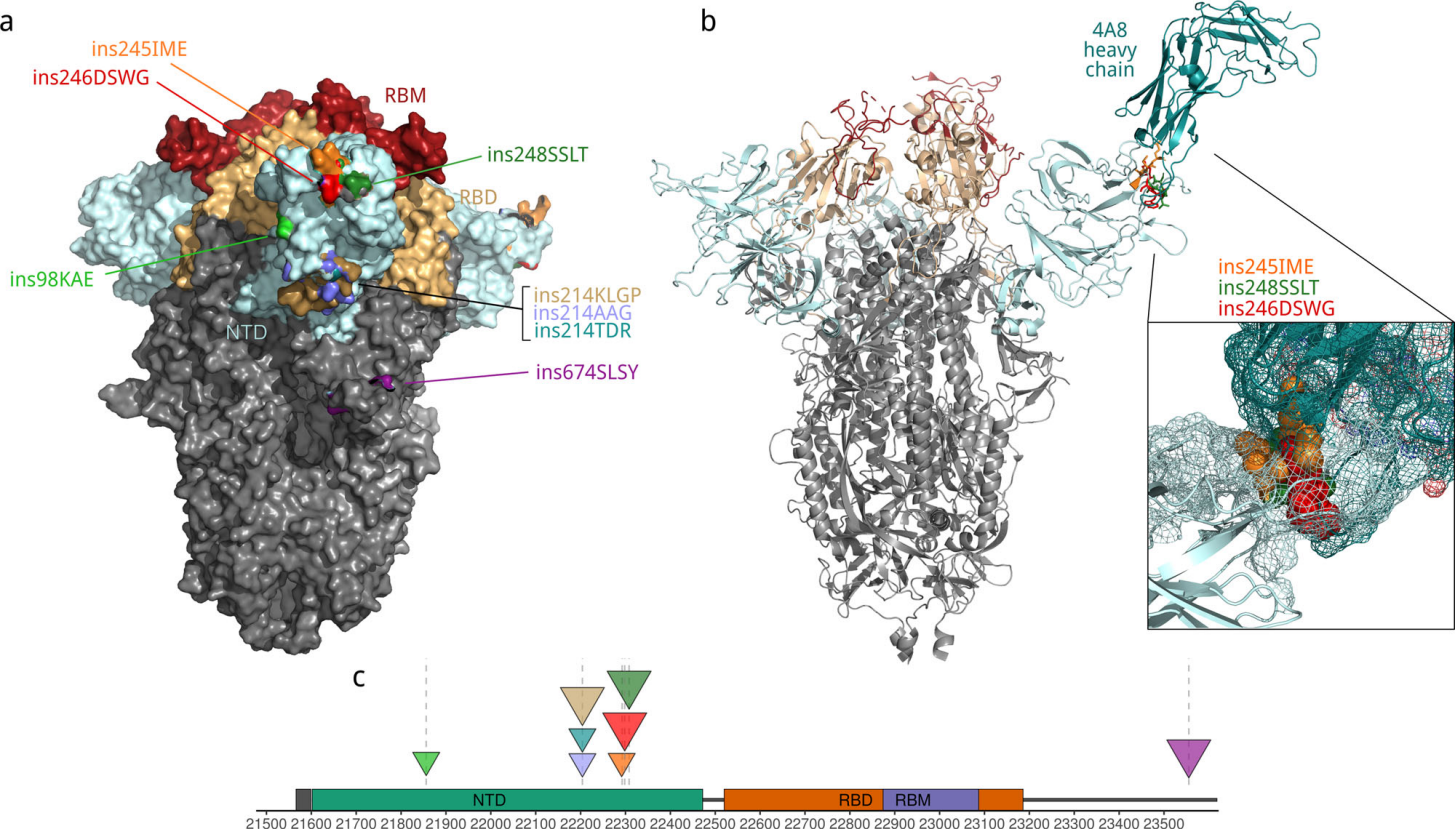
Location of insertion sites in SARS-CoV-2 S protein. **a** Surface representation showing that all observed insertions can potentially change surface properties (PDB ID: 7cn8). **b** Ins 245, 246 and 248 are located on the surface interacting with 4A8 antibody (PDB ID: 7cl2). Enlarged is the interacting surface. Cyan, N-terminal domain (NTD), wheat, receptor-binding domain (RBD), dark red, receptor-binding motif (RBM), aquamarine, heavy chain of the 4A8 antibody (PDB ID: 7cl2). Each insertion is shown in a distinct color. The models for each insertion were generated with the SWISS-model web server. **c** Location of insertions in the genome of SARS-CoV-2. Full description of insertions is provided in the Supplementary Data 4. Triangle size is proportional to the insert length.

All eight long inserts in S protein were located on the surface, within computationally predicted epitope candidates, and could potentially modify the interactions of S with the receptor and/or antibodies^36^ (Fig. 3a, b). Seven of these eight inserts mapped to the NTD of S, and three of these occurred in the same genome position, 22,004 (Fig. 3c). Compared to the receptor-binding domain, the NTD initially attracted much less attention. Subsequently, however, multiple substitutions associated with variants of concern and observed in immunocompromised individuals with extended COVID-19 disease were identified in the NTD^2,40,41^. To evaluate potential functional effects of the inserts in the NTD, we mapped them onto the protein structure. Three inserts, ins245IME, ins246DSWG and ins248SSLT, are located in the loop that is responsible for the interaction with the 4A8 antibody and potentially other antibodies^42^ (Fig. 3). Thus, at least these three insertions might be associated with the escape of SARS-CoV-2 variants from immune antibodies. The presence of multiple insertions in the same site 22,004 is suggestive of a role in infection, which is compatible with the observation of multiple deletion variants in the same region, in particular 21,971–22,005^10^. These insertions and ins98KAE are located in the neighboring loops, and given that the central region of the NTD is essential for the virus interaction with CD4 + cells^43^, could be associated with the escape from the T-cell immunity. Also, there is additional evidence that this region could represent another epitope for antibody binding^44^. Although by the end of the summer of 2021 most SARS-CoV-2 variants have been supplanted eradicated by the Delta variant^45^, these insertions merit further monitoring because they were detected in recent samples, and probably can influence viral immune response. If some of those variants will recombine with Delta it can potentially lead to serious implications.

## Discussion

Although structural variation is an important driver of betacoronaviruses evolution, in the genome analysis during the COVID-19 pandemics, part of the structural variations, namely, long insertions, to our knowledge, have not been systematically analyzed. This is a potentially consequential omission, given that insertions in the S and N proteins might contribute to the betacoronavirus pathogenicity. In particular, the furin cleavage site inserted into the S protein seems to be crucial for SARS-CoV-2 pathogenicity^46,47^, and insertions that seem to differentiate high-pathogenicity coronaviruses from low pathogenicity ones have been detected in both S and N^11^. Furthermore, betacoronaviruses are known to produce transcripts of greater than genome length^24^, suggesting that insertions occur frequently during the reproduction of these viruses. Here, we attempted comprehensive identification and analysis of insertions in the SARS-CoV-2 protein-coding sequences that originated during the current pandemic. Although the number of inserts reported here is likely to be an underestimate because inserts are not always reported in the genome sequences (Supplementary Note 1), we were able to compile a dataset containing 346 inserts.

We found that short and longer insertions substantially differed with respect to their nucleotide compositions and mapping to the phylogenetic tree, suggesting that different mechanisms could be at play. The short inserts were strongly enriched in U and in most cases occurred independently in the tree. It appears likely that these inserts resulted from RdRP slippage on short runs of A or U. Indeed, the observed excess of U in these inserts resembles the error profile of SARS-CoV-1 RdRP^36^. In contrast, the composition of the long inserts was close to that of the virus genome, and many of these insertions were found to be monophyletic, that is, these appear to be rare events that did not occur at nucleotide runs. Sequence analysis of the SARS-CoV-2 genomes indicates that these insertions occur either through polymerase slippage resulting in tandem duplication or more commonly, seem to have been triggered by illegitimate template switching associated with the formation of nc sgRNAs. In support to our hypothesis template switching in different RNA viruses has been demonstrated previously in a variety of experimental settings^21,48^. Furthermore, template switching has been observed in coronaviruses^49^.

For approximately one third of the long insertions, we were unable to pinpoint the source of the inserted sequence. This could be explained simply by the mutational deterioration of the similarity between the source and insert sequences, especially, for 9 nt inserts. However, a third mechanism of insertion cannot be ruled out. The PRRA insert that comprises the furin cleavage site (PRRA) in the S protein resembles the long inserts that occurred during the pandemic. Although we were unable to identify a statistically significant match that would allow us to map the origin of the PRRA insert to a particular location within the SARS-CoV-2 genome, this insert also might have originated by template switch, with subsequent substitutions erasing the similarity to the origin sequence. As an alternative, the origin of this insert by template switching and recombination with another sarbecovirus remains a possibility^50^.

Long inserts are markedly overrepresented in the S glycoprotein, particularly, in the NTD. Examination of the locations of these inserts on S protein structure strongly suggests that at least some of the inserts in the NTD can influence binding of the respective variants with neutralizing antibodies and, possibly, also modify the T-cell response. The excess of insertions in the S protein is compatible with this protein being the principal area of virus adaptation. However, the location of most of the inserts in the NTD, as opposed to the RBD, appears unexpected. Considering that all the detected inserts appeared at a relatively late stage of the pandemic, it seems likely that the structure of the RBD was already largely optimized for receptor binding at the onset of the pandemic such that most insertions would have a deleterious effect. In contrast, insertions into the NTD might allow virus to escape immunity without compromising the interaction with the host cells.

Although many variants reported here are likely to be supplanted by the Delta variant that currently (October 2021) dominates the globe, some of insertions described here could contribute to the emergence of escape variants, even if these variants are likely to lose in the competition with the more contagious Delta^51–54^. The appearance of the same or similar inserts in the Delta background, possibly, by recombination, might lead to epidemiologically relevant consequences. Thus, the insertion variants appear to merit monitoring, especially, at a time when vaccination could select for escape variants.

## Methods

### GISAID data

The full multiple alignment of 1,785,103 complete SARS-CoV-2 genomes (version 0617) was downloaded from GISAID (https://www.gisaid.org/). From this alignment, we extracted all positions of insertions. An insertion was defined as addition of any number of columns compared to the SARS-CoV-2 reference genome (hCoV-19/Wuhan/Hu-1/2019 (NC_045512.2)). All insertions detected in the first and last 100 positions of the reference sequence were discarded as potentially erroneous. The alignment around the potential insertions was manually inspected, and all inserts that were the result of misalignment were discarded. All the sequences that had more than two insertions were discarded, in order to avoid genomes with multiple sequencing errors. Also all inserts with ambiguous symbols were discarded. Information on the laboratory of origin, sequencing platform and consensus assembly methods (where available) was extracted from GISAID metadata. In addition, because GISAID removes from alignment all inserts >12 nucleotides that appeared only once, we downloaded the metadata for all genomes with inserts, that were submitted before 2021-06-24, and selected those events that were at least 12 nucleotides long. We retained only those that had corresponding data in SRA, and were confirmed by raw read data analysis (see below).

To identify all genomes that contained the particular insert, we downloaded all GISAID sequences available by 2021-06-23 and used cdhit-est-2d to find identical sequences with parameters: -s 0.99 -c 1.0 -n 11.

### Insertion validation from raw read data

Raw reads were downloaded from SRA database (https://www.ncbi.nlm.nih.gov/sra) with SRA Toolkit (Supplementary Data 1). The reads were mapped to the SARS-CoV-2 reference genome (NC_045512.2) with bowtie2 version 2.2.1^55^, either in pair mode of single read mode, depending on the type of data deposited to the SRA. The variants in each genome were called with LoFreq version 2.1.5^56^ as described in Galaxy (https://github.com/galaxyproject/SARS-CoV-2/blob/master/genomics/4-Variation/variation_analysis.ipynb). All insertions identified with LoFreq were visualized with the IGV software and manually inspected. An insertion was considered a real biological event if it had an allele frequency in reads more than 50%, was located in the middle of the amplification fragment, and was covered by at least ten reads.

### Search for origins of long insertions

Search for putative duplications/template switch events with and without mismatches was performed against SARS-CoV-2 and closely related SARS-CoV genomes from human and 43 bats (https://hgdownload.soe.ucsc.edu/goldenPath/wuhCor1/multiz44way/). A pangolin SARS-CoV (MT040335.1) was added to this dataset. Each insertion sequence was compared to all subsequences from a target sequence. All sequences with either the perfect match or with mismatches was retrieved (putative insertion source, PIS). If a PIS was located immediately upstream or downstream of an insertion sequence, it was annotated as duplication. If the PIS was located in any other positions, the template switch model was accepted as the best explanation of the observed insertion sequence. This procedure was implemented using a sliding window, of a length equal to the length of the analyzed insert. If no perfect match was detected, the window with the minimal number of mismatches was retrieved and considered a putative insertion source.

To assess the significance of putative duplications and template switch events, we designed a sampling procedure to test a hypothesis that an insertion is not the result of spurious matches between an insertion sequence and corresponding PIS. Each insertion sequence was shuffled and scanned against datasets using the sliding window described above. This procedure was implemented as a set of C++ and Fortran programs (see Code Availability). Manual inspection of results was performed using the FASTA3 program^57^. The number of mismatches between an insertion sequence (observed or shuffled) and PIS was taken as weight W. The distribution of weights W_shuffled_ was calculated for 1000 shuffled insertion sequences This distribution was used to calculate probability P(W_observed_ ≥ W_shuffled_). This probability is equal to the number of shuffled insertion sequences with W_shuffled_ equal to or smaller than W_observed_. Small probability values (P(W_observed_ ≥ W_shuffled_) ≤ 0.05) indicate statistical support for the hypothesis that the analyzed insertion sequence results from a duplication or a template switch.

Short inserts (<9 nucleotides) were marked as a duplication if the insert was identical either to the left or the right adjoining sequence. In addition, we separated single-nucleotide inserts occurring within homopolymer runs from the rest of the inserts.

### Analysis of transcriptome data and genomic RNA structure

To compare insert locations with RNA secondary structure, we utilized the data from Huston et al., 2021 uploaded to github: https://github.com/pylelab/SARS-CoV-2_SHAPE_MaP_structure. For our analysis we used the data from full-length secondary structure map (.ct file). We considered all paired bases to be in stems, whereas those that are not paired were considered to be located in the loops. Thus, an insert was assigned to the stem if it was flanked on both sides by residues known to be paired.

The data on the SARS-CoV-2 transcriptome was extracted from ref. ^26^. Pearson correlation coefficient between insertion locations and template switch hotspots was calculated for bins of size 100 nucleotides with cor.test() function in R version 3.6.3.

To calculate the random distributions for the analyses of distances to the closest junction and appearance of insertions in stems, we performed 1000 permutations, where each time we selected randomly from genome the same number of genome positions as in inserts dataset (347 when we analyzed all inserts, and 45 when we analyzed highly confident inserts). To compare distribution of distances for real data and random data, the Wilcoxon sum rank test was performed. In the case of inserts in stems, the *p* value is the portion of cases in our simulation that had the same or smaller number of junctions as the real data.

To analyze whether long insertions coincide with template switch hotspots, we utilized the data on 5′ and 3′ ends of junctions from^26^. All junction reads within 100 nucleotides from insertion site and insertion source positions were selected. Although the core elements that are involved in template switching are 6–7 nt long, the neighboring regions appear to contribute as well^23,58^. Furthermore, the peak areas representing the hotspots in the original data are wide, and in the initial publication, 100 nt windows were used for the analysis (Fig. 1e)^26^. To verify the significance of these findings we performed two simulations. In first scenario the positions of inserts were fixed to the real positions from the data, but the locations of source sequences were randomly sampled 1000 times from the genome, in second scenario both source and insertion site positions were randomly sampled 1000 times. The *p* value is the portion of cases in our simulation that have the same or larger number of junctions as the real data.

### Phylogenetic analysis

To find the location of the selected SARS-CoV-2 genomes on the phylogenetic tree we utilized UShER^59^. The phylogenetic tree of 2,501,152 genomes from GISAID, Genbank, COG-UK and CNCB (2021-07-16) available at UCSC was used as the starting tree, and only leaves representing records from GISAID (1347414 leaves) retained.

To assign PANGO lineages to genomes, we utilized pangolin software v. 3.1.5^60^ with default parameters.

An insert was defined as strictly monophyletic if it was observed in at least two genomes, and those genomes formed a stable clade in the phylogenetic tree or were located in the same stem cluster (set of identical sequences in the tree with branches of zero length). Also. we used more relaxed criteria, which implies that inserts in clades that contained less than 1.5% of all genomes in the dataset (2000 leaves) and belonged to the same PANGO lineage were likely monophyletic.

The clades containing the genomes of interest were extracted and vizualized with ETE 3 package for Python^61^.

### Models of spike protein and visualization

Models were build with SWISS-model^62^. We used the basic parameters. The models shown on Fig. 3 are based on two different initial PDB structures: Cryo-EM structure of PCoV_GX spike glycoprotein (PDB ID: 7cn8), and complex of SARS-CoV-2 spike glycoprotein with 4A8 antibody (PDB ID: 7cl2). The first structure was selected, because it was the best whole length structure with highest aa identity, that cover most of the protein.

The obtained protein models were visualized with Open-Source PyMOL version 2.4.

### Statistics and reproducibility

All statistical analysis was performed in R v. 3.6.3, and all corresponding scripts are provided (see Code availability). To analyze correlations between transcriptome data and positions of inserts, Pearson correlation coefficient was calculated for bins 100nt long (299 bins in each category). Distance from each insert to the closest junction or location of insertion in a stem was calculated either for all inserts (347 events) or for high-confidence inserts (45 events). Background distributions were produced by random sampling of the same number of positions as in the test dataset from a genome of the same size. The significance of the difference between the background and observed distributions was calculated as either Wilcoxon rank sum test *p* value or permutation test *p* value. To confirm the significance of the identified origins of inserts, the permutation test was performed as described under Methods. The co-occurrence of template switches with recombination hotspots was analyzed for 29 long high-confidence inserts, and the significance was estimated as permutation test *p* value (see Methods) with 10,000 permutations.

### Reporting summary

Further information on research design is available in the Nature Research Reporting Summary linked to this article.

## Supplementary information

Supplementary Information

Description of Additional Supplementary Files

Supplementary Data 1

Supplementary Data 2

Supplementary Data 3

Supplementary Data 4

Supplementary Data 5

Reporting Summary

## Data Availability

GISAID data used for this research are subject to GISAID’s Terms and Conditions. SARS-CoV-2 genome sequences and metadata are available for download from GISAID EpiCoV^™^. The acknowledgements to all originating and submitting laboratories are provided in the Supplementary Table 5. Source data underlying Figs. 1–2 is presented in Supplementary Data 2.

## Code availability

Custom R and Python scripts utilized for data analysis and visualization are available on github: https://github.com/garushyants/covid_insertions_paper and published on Zenodo with 10.5281/zenodo.5567908^63^.
