# Supplementary material for: Template switching and duplications in SARS-CoV-2 genomes give rise to insertion variants that merit monitoring
Source: Commun Biol. 2021 Nov 30;4:1343. doi: 10.1038/s42003-021-02858-9 (PMC8632935; doi:10.1038/s42003-021-02858-9)
Supplement: Supplementary file 2 — Description of Additional Supplementary Files [file 42003_2021_2858_MOESM2_ESM.pdf]

Description of Additional Supplementary Files

File name: Supplementary Data 1

Description: The list of inserts in SARS-CoV-2 genomes and genomes where they were found. Abbreviations in LoFreq Info column: DP – Raw Depth, AF – Allele Frequency, SB – Phred-scaled strand bias at this position, DP4 – Counts for ref-forward bases, ref-reverse, alt-forward and alt-reverse bases, HRUN – Homopolymer length to the right of report indel position.

File name: Supplementary Data 2

Description: The summary of supporting data for 354 inserts observed in this study. The details on the monophyly test are provided in Materials and Methods.

File name: Supplementary Data 3

Description: SRA records information for genomes found on the same clade as sequences containing ins22204:ATAGATCGA and ins22205:CGGCAGGCT.

File name: Supplementary Data 4

Description: The list of high confidence inserts with the potential mechanisms of their appearance. For template switch candidate the location of origin, sequence in origin and p-value are specified.

File name: Supplementary Data 5

Description: The acknowledgments to all Originating and Submitting laboratories that provided the data to GISAID utilized in this paper.
